# Persistent detection of Tilapia lake virus in wild tilapia and tinfoil barbs

**DOI:** 10.14202/vetworld.2022.1097-1106

**Published:** 2022-04-27

**Authors:** Azila Abdullah, Afzan Muntaziana Mohd Pazai, Mohd Syafiq Mohammad Ridzuan, Fahmi Sudirwan, Shahidan Hashim, Adnan Abas, Munira Murni, Zuraidah Roli, Rimatulhana Ramly, Mohd Firdaus-Nawi

**Affiliations:** 1 National Fish Health Research Division (NaFisH), Fisheries Research Institute (FRI) Batu Maung, Department of Fisheries Malaysia,11960 Batu Maung, Penang, Malaysia; 2 Freshwater Aquaculture Fisheries Research Division, Fisheries Research Institute (FRI) Glami Lemi, Department of Fisheries Malaysia, 71650 Titi Jelebu, Negeri Sembilan, Malaysia; 3 Perlis State Fisheries Department, Department of Fisheries Malaysia, Lot 636 Kuala Perlis Road, 02000 Perlis, Malaysia; 4 Department of Marine Science, Kulliyyah of Science, International Islamic University Malaysia, 25200 Kuantan, Pahang, Malaysia

**Keywords:** Malaysia, Tilapia lake virus, tinfoil barbs, wild tilapia

## Abstract

**Background and Aim:**

One of the emerging viral diseases in freshwater fish is Tilapia lake virus (TiLV), which infects all stages of fish and results in mass mortalities. Previously, a TiLV case was detected in the wild environment in Malaysia that involved tilapia and tinfoil barb. Hence, this study aimed to determine the presence of TiLV in wild tilapia (*Oreochromis niloticus*) as well as tinfoil barbs (*Barbonymus schwanenfeldii*) at the similar lake after the initial outbreak in year 2017.

**Materials and Methods:**

Both fish species were sampled from this lake at a month interval for two years and subjected to TiLV detection using reverse transcriptase-polymerase chain reaction and cell culture isolation. Concurrently, bacterial isolation and water quality measurements were performed to deduce their correlation with TiLV occurrence. Other wild fish species and mollusk were also occasionally sampled during the fish inventory activity at this lake. The fish’s weight, length, and associated clinical signs were noted throughout the entire study period.

**Results:**

Mortality was not observed throughout the whole study period, and results indicated a moderate to high prevalence of TiLV infection in both tilapia and tinfoil barbs. There was no correlation between TiLV infection with the isolation rate of opportunistic bacteria such as *Aeromonas spp., Plesiomonas spp*., and *Edwardsiella spp*. in the study site. At the same time, the Pearson correlation test revealed a moderate negative correlation between the water pH with the presence of TiLV (R=−0.4472; p<0.05) and a moderate positive correlation between the water iron content with the monthly detection of *Aeromonas spp*. in wild tilapia. This is contrary to tinfoil barbs, where there was a moderate negative correlation between the water iron content with the monthly isolation of *Aeromonas spp*. (R=−0.5190; p<0.05). Furthermore, isolation of TiLV on cell culture-induced viral invasion was resulted in the cytopathic effects.

**Conclusions:**

Our results suggest that the wild fish may harbor TiLV for an extended period following a massive die-off event in 2017 without any obvious clinical signs and mortality. The persistency of viruses in the wild may need continuous and effective control as well as prevention strategies.

## Introduction

Tilapia lake virus (TiLV) is a virus that belongs to the Amnoonviridae family and is a member of the recently established Tilapinevirus genus [1]. It is a newly emerging virus that affects the population of tilapia species globally. The disease was first detected in Israel during the summer of 2009, where many fish farms recorded significant losses of tilapia from May to October [2]. The disease affected farms that cultivated Nile Tilapia (*Oreochromis niloticus*), red tilapia (*Oreochromis* spp.), and hybrid tilapia (*O. niloticus×Oreochromis aureus*), where it caused 20-90% of mortality [2-6]. Alarmingly, the virus’s susceptibility has been demonstrated in a variety of hosts, including giant gourami [7], ornamental African cichlids [8], Mozambique tilapia [9], and zebrafish [10].

Outbreaks and experimental studies revealed that TiLV commonly affects the early stages of tilapia, including in fertilized eggs, yolk-sac larvae, fry, and fingerlings [11-14], with high mortalities among fish weighing 1-50 g when their immunity is relatively low [6]. Till now, at least 16 tilapia-producing countries have been affected by this virus [15]. The clinical signs observed in infected fish include body discoloration, skin erosions, scale loss, moderate congestion of spleen and kidney as well as lesions of the brain such as edema, focal hemorrhages in the leptomeninges, exophthalmia, abdominal swelling, loss of appetite, gathering at the bottom, slow movement, and stop schooling before death [2,13,16,17]. The risk factors include stress-associated [5], but no studies have provided evidence on other water physicochemical risk factors such as temperature, salinity, pH, and dissolved oxygen (DO). TiLV did not only affect farmed tilapia, but also it began in the wild environment of Lake Victoria, suggesting that this disease can be found anywhere in tilapia environment [2,17]. There was a case where TiLV was detected in wild tilapia and tinfoil barb from a man-made lake in Malaysia [18,19]. The reason for this finding is yet to be determined. Lakes in Malaysia play essential roles as the state’s reservoir for irrigation of agricultural activities, for aquaculture, as a source of drinking water, for prevention of seasonal floods, and last but not least, as a tourism center. It is also a place for the restocking activity of the local fish species such as tinfoil barbs (*Barbonymus schwanenfeldii*), *Hampala macrolepidota*, *Osteochilus hasselti*, *Barbonymus gonionotus* (Javanese carp), and also freshwater shrimp.

In aquaculture, multiple factors may cause the disease to emerge. These factors are typically associated with the environment, including water quality and seasonal variation, pathogenic agents, and host factors. In wild fishes, however, due to the diversity of species and lower population rate, the disease usually occurs without notification, and the cross-infection between species is seldom reported. However, for TiLV, the first observed occurrence of this disease in a wild environment had been reported where two species were involved [18].

Thus, this study aimed to determine the presence of TiLV in the same lake after the initial outbreak involving both fish species, tilapia (*O. niloticus*) and tinfoil barbs (*B. schwanenfeldii*). Concurrently, bacterial isolation and water quality monitoring were performed to deduce their correlation with TiLV and the possibilities of transmission to/from other wild fishes and mollusk species caught from this lake. In addition, establishing the risk factors involved in this disease is vital for effective control and prevention of the disease in a wild environment.

## Materials and Methods

### Ethical approval

The handling of fish and all experimental procedures in this work was performed per the guidelines of animal usage for scientific purposes from The Institutional Animal Care and Use Committee (I-ACUC), International Islamic University Malaysia, as certified in permission number IACUC-2020-015.

### Study period and site

The sampling was conducted for a 2-years period from March 2018 to March 2020 at a monthly interval without fail and conducted at a lake longitude 100° 14’ 13.2” E and latitude 6° 35’ 09.9” N, in the northern part of Peninsular Malaysia. Previously, an event of mass mortality was encountered in this lake and was associated with TiLV infection [18]. The lake mainly functions to prevent flood, besides acting as a reservoir for agriculture irrigation, a tourist attraction, and a fishing ground. The lake has also been designated as one of the favorite places for migratory birds. The lake size is about 1300 hectares and can hold about 35-40 million liters of water. The state is known to have quite a distinct hot and cold season compared to the other states in Malaysia. The highest temperature recorded was up to 38.2°C (mean=28-31°C) and the lowest was 21.9°C (mean=23-27°C) during the study period (data obtained from State’s Meteorological Department). The rainfall was scarce during the hot and dry season but ranged between 0 and 41.8 mm during the windy season. Thus, the water level at this lake was usually influenced by these seasons.

### Fish samples collection

Two wild fish species that were obtained from local inland fishermen were sampled: Tilapia (*O. niloticus*) and tinfoil barbs (*B. schwanenfeldii)*. The fish were kept alive in a tank supplied with an aerator until the postmortem *in situ*. Each fish’s length and weight were measured, and after that, any external gross lesions as well as clinical signs were recorded accordingly. Mollusk samples were collected four times along the sampling duration as supportive findings. During the fish inventory activity by the lake’s authority, several other freshwater fish species were also collected and sampled from the study sites. We also managed to catch smaller size fish (<4 cm) of tilapia and tinfoil barb using a net of smaller size around the lakeside. The details of the type of samples are shown in Table-1.

**Table 1:** Description of samples collected from the sampling site between March 2018 and March 2020.

Description	Tilapia	Tinfoil barbs
Number of fish sampled	291	475
Size (g)	1-1850	1-750
Number of bacteriology samples	873	1425
Number of PCR samples	246	232
Number of tissue culture tests	46	16
Organ samples for bacteriology	Brain, eye and kidney	Liver, kidney and spleen
Mollusc samples	i. *Unionetta* *fabagina* ii. *Corbicolla* *fluminea* iii. *Pila* *ampullaceal* iv. *Filapaludina* *martensi*	
Other wild fish species	i. *Cichla ocellaris* (7 fish) ii. *Barbonymus gonionathus* (3 fish) iii. *Oxyeleotris marmorata* (5 fish) iv. *Geophagus altifrons* (10 fish) v. *Pristolepis fasciata* (4 fish)	
Water quality parameters	Dissolved oxygen, pH, temperature, ammonia, nitrite, sulfate, iron	

PCR=Polymerase chain reaction

### Water quality measurement

The water physicochemical parameters were collected at three different points, specifically at the inlet, middle, and outlet of the lake. At each sampling point, the water was collected at two different depths, which were surface and bottom depth, depending on the water level. The maximum water level recorded was 4 m and the minimum was a 1-m depth. The physical parameters were measured onsite, around noon (12-1 pm, Malaysia time). The parameters measured included pH, temperature, and DO, which were measured using a hand-held YSI meter (YSI Inc., Yellow Springs, Ohio, USA). The concentrations of nitrite, sulfide, iron, and ammonia were then determined using powder pillow procedures and measured with a spectrophotometer (HACH Company, Loveland, Colorado, USA). Daily temperature and rainfall data were also obtained from the State Meteorology Department for data analysis comparison.

### Bacterial isolation and identification

The collected fish were euthanized with diluted Tricaine Methanesulfonate (MS-222) at 250 mg/L. Brain, eye, and kidney swabs of tilapia were aseptically streaked onto blood agar and kept at ambient temperature on arrival to the lab. Meanwhile, swab samples of liver, kidney, and spleen of tinfoil barbs and other species were streaked on the Tryptic Soy Agar (Merck, Darmstadt, Germany) and kept in the same condition. Subculture was done by selecting the dominantly grown colony to get pure colonies before they were divided into different types according to the colony characteristics [20]. Pure isolates were subjected to Gram-staining before biochemical tests. Finally, identification of the species was made using the established protocol from the API test kit (bioMérieux, Marcy I’Etoile, France).

### Sample collection for virus detection

Fish that were more than 4 cm in length were dissected. Fish organs such as liver, kidney, and spleen were pooled and kept in viral transport media (VTM), which consisted of Hank’s balanced salt solution (HBSS), 2% fetal bovine serum, and 0.05 mg/mL gentamicin-sulfate (Thermo Fisher Scientific, Waltham, Massachusetts, USA) in 15 mL tubes. Smaller-sized fish were collected as whole fish and pooled with 2-5 fish in VTM. These samples were kept on ice until they arrived at the laboratory and processed within 36-48 h. Besides, the samples from other wild fish species during the inventory activity were kept in Ribonucleic acid later (RNAlater™, Invitrogen, USA) at 4°C until analysis.

### Cell culture isolation

At the laboratory, samples in VTM were homogenized with cold mortar and pestle and added with 10 mL fresh HBSS containing 2% fetal calf serum and 0.05 mg/mL gentamicin-sulfate. The mixture was then centrifuged at 4000× *g* for 10 min (Sigma 2-15k Sartorius, Goettingen, Germany), and the supernatants were filtered with 0.2 μm syringe filters. The filtered samples were stored at 4°C for cell culture inoculation or at −80°C for polymerase chain reaction (PCR) analysis [20].

A total of 200 μL of filtered supernatant were inoculated into a 24-well plate (Thermo Fisher Scientific) containing a monolayer of E-11 cells. The media were first discarded and washed once with HBSS before inoculation of the samples. The culture was then incubated for 1 h at 25°C before the fresh L-15 medium (Thermo Fisher Scientific) supplemented with 2% fetal calf serum and gentamicin 0.05 mg/mL was added. The plate was further incubated at 25°C, and the formation of cytopathic effect (CPE) was observed daily until all cells detached. After that, this CPE will become a blind passage into new E-11 cells for at least three passages. Any CPE formed after the third blind passage was harvested and tested in the subsequent reverse transcriptase-PCR (RT-PCR) technique for confirmation.

### RT-PCR

Total RNA from the above-filtered supernatant was extracted using TRIzol™ LS reagent (Thermo Fisher Scientific). A set of new primers targeting segment 3 of TiLV was designed using National Centre Biotechnology Institute software. The primers were constructed using our local isolate sequences that were previously obtained from Malaysian outbreak cases.

The primer sequences were named as F3-MalTiLV (5’ TGGGCACAAGGCATCCTAC 3’) and R3-MalTiLV (5’ CACGTGCGTACTCGTTCAGT 3’). 3 μL of the extracted RNA was used as a single-step PCR template using the MyTaq™ One-Step RT-PCR kit (Meridian Bioscience, Memphis, Tennessee, USA). PCR amplifications were performed using T1000 Touch™ thermal cycler (Bio-Rad Laboratories Inc., Hercules, California, USA) as follows: Reverse transcription at 45°C for 30 min, polymerase activation at 95°C for 60 s followed by 30 cycles of denaturation at 94°C for 10 s, annealing at 55°C for 10 s, extension at 72°C for 30 s, and final extension at 72°C for 5 min. The PCR products were then analyzed via electrophoresis in 1.5% agarose gel and stained with RedSafe™ (iNtRON Biotechnology Inc., Gyeonggi-do, Korea). The results were viewed with a bio-imaging system (Syngene, Cambridge, UK), and the expected amplicon of 245 bp indicated successful amplification. Our laboratory used this new set of designed primers for TiLV detection.

### Gel purification, sequencing, and phylogenetic tree

Positive RT-PCR from CPE formation in E-11 cells consisting of three and 11 samples of tilapia and tinfoil barb, respectively, was further purified using innuPREP Deoxyribonucleic acid (DNA) Mini Kit (Analytik Jena GmbH, Konrad-Zuse-Strasse 1, Germany) according to the manufacturer’s protocol. All these samples were sent for DNA sequencing and the results were compared with the known sequences in the GenBank database using Nucleotide Basic Local Alignment Search Tool program. The construction of phylogenetic trees was conducted by matching the nucleotides of the isolates from tilapia and tinfoil barb with four nucleotides of segment-3 from other countries, that is, Israel [2], Thailand [13], India [21], and Ecuador [22]. The nucleotide sequence of the gene encoding for the viral coat protein of red-spotted grouper nervous necrosis virus was used to compare the genus. Evolutionary analyses were conducted in MEGA7 [23].

### Reporting of positive findings

The positive detection of TiLV was calculated as:


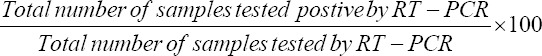


As for the pool samples, the data were initially subjected to the prevalence calculator (AusVet – https://epitools.ausvet.com.au/trueprevalence), and the results were keyed in an Excel spreadsheet.

### Statistical analysis

The mean of the water quality parameters and the monthly detection of bacterial and TiLV isolated in both tilapia and tinfoil barb were calculated. Using Statistix version 10 (Analytical Software, Tallahassee, Florida, USA), the mean prevalence of all isolated pathogens was compared using the analysis of variance coupled with the Tukey LSD All-Pairwise Comparison Test.

The degree of correlation between water physiochemical parameters and bacteria and TiLV isolation rate in wild tilapia and tinfoil barbs was determined using Pearson correlation. In this study, a perfect linear (positive) correlation was denoted by +1, whereas an inverse (negative) correlation was represented by −1. The following criteria were used to interpret correlation strength: 0.00-0.39 for no to weak correlation, 0.40-0.69 for moderate correlation, and 0.70-1.00 for strong correlation [20].

## Results

### Clinical signs and gross lesions

**Figure-1:**
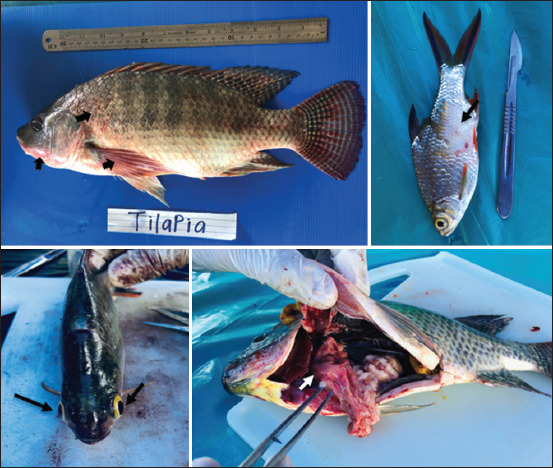
Some of the clinical signs observed in infected wild tilapia and tinfoil barbs showed body discoloration (bold arrow), scale drop (thin arrow), eye abnormalities or congested (black arrow), and pale and patchy liver (white arrow).

Externally, 98/291 or 34% of tilapia showed body discoloration, ulceration, and fin rot. In contrast, less severe lesions were observed in tinfoil barbs, with 76/475 or 16% showing signs of body discoloration and tail/fin rot. Internally, 138 (47%) tilapias showed several abnormalities, including congested kidney (34%), soft and watery brain, dark liver, enlarged spleen, and pale liver. Meanwhile, 46.7% of tinfoil barb showed signs of congested kidney and liver (57%), besides spotted black or white nodules on the liver, kidney, or spleen (Figure-1). There were no clinical signs found in other wild fishes and mollusk species. Two of these wild fish species, *Cichla ocellaris* and *Geopaghus altifrons*, are regarded as alien species and have dominated this lake for quite some time. In addition, no mortality of wild tilapia and tinfoil barbs was observed throughout the study period.

### Isolation rates of *Aeromonas* spp., *Plesiomonas* spp., *Edwardsiella* spp., and TiLV

The most frequently identified bacteria in wild tilapia were *Aeromonas* spp. and *Plesiomonas* spp., with mean detection rates of 10.61% and 4.2%, respectively. Meanwhile, in wild tinfoil barb, *Edwardsiella* spp. was also frequently isolated in addition to the above two bacteria. *Aeromonas*, *Plesiomonas*, and *Edwardsiella* spp. were detected in tinfoil barbs with mean detection rates of 35.85%, 8.21%, and 4.72%, respectively. Surprisingly, the detection of TiLV in tinfoil barbs (46.99%) was slightly higher than tilapia (32.16%), indicating that the tinfoil barbs were more susceptible to TiLV. There was a significant difference (p<0.05) in the rate of *Aeromonas* spp. isolation between tilapia and tinfoil barbs and between the different types of pathogen in tilapia and tinfoil barbs (Table-2).

**Table 2:** The mean comparison and detection rate of bacteria and Tilapia lake virus in wild tilapia and tinfoil barb.

Species	*Aeromonas* spp. (%)	*Edwardsiella* spp. (%)	*Plesiomonas* spp. (%)	TiLV (%)
Tilapia	10.61±19.65^b^	-	4.20±7.94^b^	32.16±31.90^a^
Tinfoil barb	35.85±17.15^a^	4.72±7.28^b^	8.21±8.53^b^	46.99±30.33^a^

^a,b^ Different superscript letters represent a significant difference (p<0.05) between the same row, TiLV=Tilapia lake virus

### Isolation pattern of *Aeromonas* spp., *Plesiomonas* spp., and TiLV in wild tilapia

**Figure-2:**
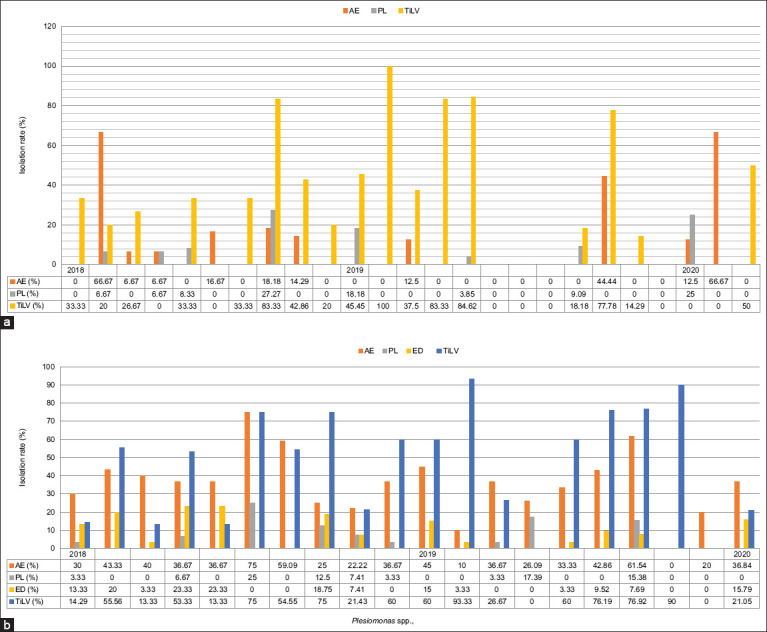
Monthly isolation of AE, PL, ED, and TiLV in wild (a) tilapia and (b) tinfoil barbs throughout the 2 years of sampling period. AE=*Aeromonas* spp., PL=*Plesiomonas spp*., ED=*Edwardsiella* spp., TiLV=Tilapia lake virus.

The monthly isolation rates of *Aeromonas* spp., *Plesiomonas* spp., and TiLV in wild tilapia are presented in Figure-2a. There was high isolation of *Aeromonas* spp. in April 2018 and February 2020 at >60%, while the prevalence of *Plesiomonas* spp. was highest in October 2018 and January 2020 at ≥25%. On the other hand, TiLV was detected almost throughout the entire study period, with the highest rate of >80% in October 2018, February 2019, April 2019, and May 2019.

### Isolation pattern of *Aeromonas*, *Plesiomonas*, *Edwardsiella* spp., and TiLV in wild tinfoil barbs

The monthly isolation rates of *Aeromonas* spp., *Plesiomonas* spp., *Edwardsiella* spp., and TiLV in wild tinfoil barbs are presented in Figure-2b. There was high isolation of *Aeromonas* spp. in August 2018 and September 2019 at >60%, while the prevalence of *Plesiomonas* spp. and *Edwardsiella* spp. was highest in June and July as well as August 2018 with 23% and 25%, respectively. TiLV was also frequently detected in wild tinfoil barbs without an obvious pattern throughout the 2 years of sampling. The highest rate of identification of TiLV was recorded in February and October 2019 (≥90%).

### Total body weight and length of TiLV-infected wild tilapia and tinfoil barbs

**Figure-3:**
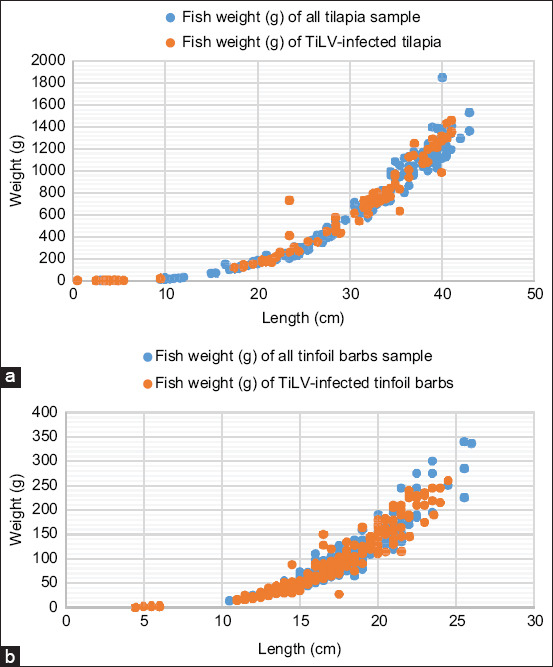
The range of weight and length of wild (a) tilapia and (b) tinfoil barbs that were infected with TiLV. TiLV=Tilapia lake virus.

Figure-3 shows the range of the total body weight and length of infected wild tilapia and tinfoil barbs from the present study. Tilapia infected with TiLV had weight that ranged between 1 g and 1460 g and length ranged between 0.5 cm and 41 cm. Similarly, tinfoil barbs infected with TiLV had weight ranging between 1 g and 260 g and length ranging between 4.5 cm and 24.5 cm. Besides, the highest frequency of positive detection was observed from smaller-sized fish of tilapia and tinfoil barbs. An attempt to correlate the fish length, weight, and prevalence of TiLV resulted in a low correlation (data not shown) for both fish species. This finding, however, indicates that TiLV can infect both fish species from a relatively early stage to adult fish.

### Water physiochemical parameters and correlation with the presence of bacteria and TiLV

The mean±SD, minimum and maximum readings of several water quality parameters are presented in Table-3. The means for water temperature, DO, and pH were 29.86±1.40°C, 6.32±1.32 mg/L, and 7.88±0.52, respectively. In contrast, the water nutrient content of nitrite, ammonia, sulfate, and iron was 0.005±0.004 mg/L, 0.018±0.017 mg/L, 10.197±6.929 μg/L, and 0.172±0.152 mg/L, respectively. The mean values of these water quality parameters fell within the normal range. However, there was some conflict on the DO, whereby the range of minimum (3.66 mg/L) and maximum (8.32 mg/L) values differed.

**Table 3:** The mean±SD, minimum, and maximum reading of the water physiochemical parameters measured at three different points during the study period.

Parameter	Temperature (°C)	Dissolved oxygen (mg/L)	pH	Nitrite (mg/L)	Ammonia (mg/L)*^1^*	Sulfate (µg/L)	Iron (mg/L)
Mean±SD	29.86±1.40	6.32±1.32	7.88±0.52	0.005±0.004	0.018±0.017	10.197±6.929	0.172±0.152
Minimum	27.00	3.66	5.91	0.002	0.000	0.000	0.023
Maximum	32.58	8.32	8.49	0.018	0.064	28.00	0.750

1 Values represent calculated un-ionized ammonia, SD=Standard deviation

Moreover, the Pearson correlation test revealed that there was a moderate negative correlation between the water pH and the presence of TiLV (R=−0.4472; p<0.05) as well as a moderate positive correlation between water iron content and the monthly detection of *Aeromonas* spp. in wild tilapia (Table-4). This is contrary to tinfoil barbs, where there was a moderate negative correlation between the water iron content and the monthly isolation of *Aeromonas* spp. (R=−0.5190; p<0.05).

**Table 4:** Correlation between water physiochemical parameters and the monthly presence of bacteria and TiLV in wild tilapia and tinfoil barbs.

Fish	WQ/Pathogen	Temp.	DO	pH	NO_2_	NH_3_	S	Fe	TiLV	AE	PL	ED
Wild tilapia	Temp.	1.0000										
	DO	0.3110	1.0000									
	pH	0.4371	0.2640	1.0000								
	NO_2_	−0.1801	−0.0905	0.1580	1.0000							
	NH_3_	0.2024	0.2324	−0.0531	−0.6296	1.0000						
	S	0.0565	−0.0639	0.0389	−0.2846	0.5715	1.0000					
	Fe	−0.3744	−0.2567	−0.1809	0.0950	0.0867	0.4646	1.0000				
	TiLV	0.0658	−0.1206	−0.4472*^a^*	−0.3878	0.3286	−0.0390	0.0327	1.0000			
	AE	−0.2229	−0.0655	0.0955	0.2663	−0.0855	−0.0137	0.4621*^a^*	−0.0905	1.0000		
	PL	−0.2325	−0.0514	0.0651	−0.2083	−0.0162	0.2467	0.0189	0.0814	0.0368	1.0000	
Wild tinfoil barbs	Temp.	1.0000										
	DO	0.4429	1.0000									
	pH	0.5080	0.2218	1.0000								
	NO_2_	0.2071	0.0083	0.2537	1.0000							
	NH_3_	0.0413	0.2749	−0.0212	−0.1327	1.0000						
	S	−0.1206	−0.1340	0.0737	0.4328	0.3521	1.0000					
	Fe	−0.3209	−0.4491	−0.2127	0.3051	0.0857	0.5705	1.0000				
	TiLV	−0.0046	−0.0457	−0.3159	0.0697	0.0104	−0.0456	0.1327	1.0000			
	AE	0.2856	0.2981	0.4262	0.0572	−0.1071	−0.1590	−0.5190*^a^*	0.0913	1.0000		
	PL	0.0750	0.1775	0.2687	−0.1249	−0.1661	0.1851	−0.1566	−0.0383	0.0511	1.0000	
	ED	0.1272	0.1108	0.0800	−0.0723	0.0535	−0.2586	−0.3121	0.0982	0.4042	−0.1412	1.0000

TiLV=Tilapia lake virus, WQ=Water quality parameters, Temp.=Temperature, DO=Dissolved oxygen, NO_2_=Nitrite, NH_3_=Ammonia, S=Sulfate, Fe=Iron, AE=*Aeromonas* spp., PL=*Plesiomonas* spp., ED=*Edwardsiella* spp.

a Indicate statistically significant at P*<*0.05

### Isolation of TiLV on cell culture

**Figure-4:**
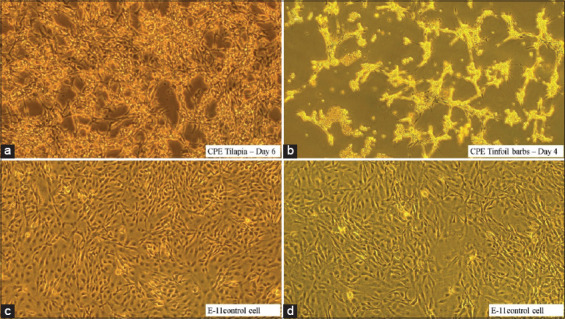
Cytopathic effect of the E-11 cells following inoculation of samples from (a) tilapia, (b) tinfoil barbs, (c and d) E-11 control cell.

The E-11 cell line obtained from NFRL, Italy, was used to isolate the virus. In tilapia, the cells usually clumped together starting from day 5 to day 7 post-inoculation, while in tinfoil barb, the CPE was much faster and started from day 3 until day 6. In tinfoil barb samples, the monolayer cells tended to shrink and disintegrate very easily compared to tilapia CPE (Figure-4).

### Phylogenetic tree analysis

**Figure-5:**
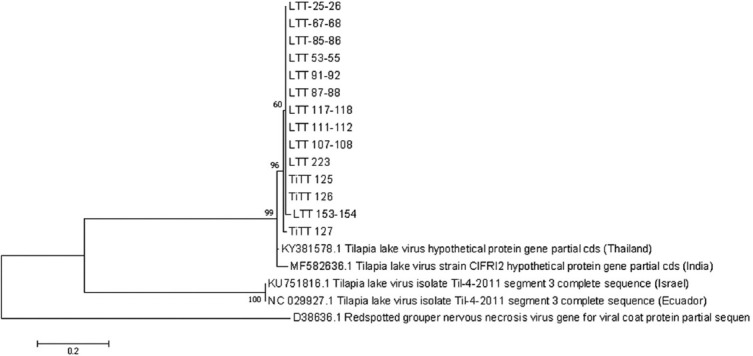
The phylogenetic tree shows the similarity of these isolates with the other countries.

The sequencing results showed that the isolated TiLV from tilapia and tinfoil barb in this study had the closest kinship with the TiLV isolate from Thailand (accession number: KY381578.1), that is, 98% similarity. Furthermore, the maximum likelihood phylogenetic tree based on the concatenated multi-locus alignment of all sequences grouped our TiLV isolates with the Thailand (KY381578.1) and India (MF 582636.1) references, respectively. In contrast, the two strains, Israel (KU157816.1) and Ecuador (NC029927.1), clustered together (Figure-5).

## Discussion

The current study was conducted after the significant mortality of tilapia and tinfoil barb at this lake was previously confirmed to be caused by the TiLV [18]. Hence, the study aimed to observe and obtain data on the reoccurrence and involvement of any risk factors of TiLV in a lake environment. The TiLV and several opportunistic bacteria species, including *Aeromonas* spp., *Plesiomonas* spp., and *Edwardsiella* spp., were consistently detected throughout the sampling period. The presence of multiple pathogens in fish might predispose them to stress. However, no distinct isolation pattern of bacteria and TiLV in wild tilapia and tinfoil barbs was observed. Therefore, we inferred that these bacteria were already established in the lake, serving as normal flora of the environment. Besides, no mortality or outbreak was observed within the study period, suggesting the existence of external factors that might influence the pathogenesis and host, which resulted in a massive die-off event in 2017. The TiLV-infected fish displayed clinical signs consistent with the previous TiLV cases such as body discoloration, skin erosion, enlarged spleen, and congestion of kidney and spleen [2,13,16,17].

Our findings demonstrated that the percentage of TiLV detection was 100% in small-sized wild tilapia and tinfoil barbs. Although the number of samples of these smaller fish was limited and only collected once during the fish inventory activity, this finding can confirm that TiLV can infect the fish at a very early developmental stage in a wild environment [24]. In addition, our results in adult fishes indicated that the positive detection of TiLV in tinfoil barb (46%) was significantly higher than tilapia (32%) and the CPE progression from tinfoil barb samples was shown to be faster in E-11 cell culture. Tinfoil barbs are members of the Cyprinidae family, which is distinct from tilapia, and they typically reside from the mid-level to the bottom of the lake. They tend to be more active compared to tilapia and prefer strong water currents. It is unclear whether these behaviors contribute to the disparate findings of faster CPE formation on cell culture and higher TiLV prevalence or whether they are a susceptible species that harbor the virus. Therefore, a detailed pathogenicity study is recommended to answer this question.

TiLV had been reported to have a concurrent infection with *Aeromonas* spp. in farmed tilapia [25,26] and more recently with a novel Tilapia parvovirus [27]. However, in a wild environment, as in this study, the correlation between the monthly isolation of bacteria and TiLV in both wild tilapia and wild tinfoil barb was insignificant. This study also provided evidence that the other wild species and mollusks that inhabit this lake were free of TiLV. Periodic sampling should be conducted to ensure this status. Mollusk can act as a viral vector as the virus can reside in the tissue for a longer time; thus, precaution should be taken by monitoring its population and its movement from this lake. Moreover, the possibility of viral spreading to other native wild fish species in the same group as tilapia and tinfoil barb may be high since TiLV had been reported to occur in giant gourami [7,28], ornamental African cichlids [8], and zebrafish [10] through experimental infection. A positive detection in healthy tilapia and tinfoil barb during this study implies that there may be an increase in the likelihood of the virus spreading to other susceptible species.

Poor water quality can aid pathogen proliferation in fish and the environment, where prolonged exposure to these suboptimal conditions can cause physical damage, behavior changes, and physiological stress of the fish, as well as suppression of the fish immune system. In this study, a moderate negative correlation between water pH and the presence of TiLV in wild tilapia was identified. However, such coincidence was absent in wild tinfoil barbs. Previously, the high mortality rates documented in Israel [2], Egypt [29], and Ecuador [5] were referred to as summer mortality syndrome due to their occurrence in the hot season, implying that increased water temperature could be a contributing factor. However, since no mortality was recorded in our observation, such a correlation cannot be established. Besides, the correlation between the monthly water temperature and the presence of TiLV was insignificant. In contrast, a significant correlation between the water iron content and the monthly detection of *Aeromonas* spp. in both wild tilapia and tinfoil barbs was discovered. Multiple infections in fish are relatively common and occur when the hosts are infected by two or more pathogens, either concurrently or secondarily, hence resulting in the coexistence of multiple infectious agents in the same host [20].

The question that stays unanswered from this study is the origin of TiLV at this lake. Malaysia usually imports tilapia from many countries for both breeding and grow-out purposes. The virus may have been introduced into this lake during the cage culture activity, pre-dated between the years from 2001 to 2010. It is important to note that cases of TiLV had been reported in Israel and other countries as early as 2005-2009 [2], whereas in Thailand, the cases were traced back to the 2000s [30]. A study on the phylogenetic tree of the virus may validate its origin; however, complete genetic sequences are needed to picture the result better. An analysis of all the ten segments showed wide sequence variations between the segments of TiLV, specifically segments five and six [30,31].

The control and prevention strategies as outlined in the OIE disease card may work very well in cultured species. However, it will be challenging to implement them in a wild and natural environment. This study provides an indication that TiLV may be persistent in wild environment and may take a longer time to diminish; therefore, restocking of fish at the same affected areas needs to be carefully reviewed to prevent further spreading. The status of TiLV should be routinely monitored before resuming stocking activities. As such, a pooling strategy of 5-10 fish could be applied in the routine sampling process during the critical periods: rainy and dry seasons or 3-4 times annually to minimize cost and workload [32]. Besides, the source and health status of the fish used in restocking activities should be verified to ensure that they come from reputable sources and are tagged with a health certificate. Vectors such as mollusks and visitors’ activities should be closely monitored and controlled to avoid invasion and the introduction of contaminated sources. Continuous surveillance of TiLV should be conducted occasionally at any connecting public water body. The functions of the lake as a large water reservoir and irrigation may be reviewed by looking at the possibilities of controlling the inlet and outlet, especially during an outbreak, with caution on the surrounding aquaculture activities. Additional research may be undertaken to deduce the pathogenicity, transmission, and risk factors of TiLV in Malaysia. The search for potential antiviral drugs, herbal remedies, or vaccines is desirable and should be explored.

## Conclusion

This study has highlighted the persistent detection of TiLV in wild tilapia and tinfoil barbs following a massive die-off event in 2017. Our results suggest that wild fish may harbor TiLV for an extended period without obvious clinical signs and mortality. The persistency of viruses in the wild may need continuous and effective control and prevention strategies. However, our study was constrained by the unequal number and size distribution of fish sampled throughout. Future research should focus on the pathogenicity of TiLV in tinfoil barbs since the highest prevalence rate was observed among these species.

## Authors’ Contributions

AA, AMMP, MSMR, FS, SH, AA, MM, ZR, and RR: Execution of the project and data analysis. AA and AMMP: Conceived and framed the main idea of this study. AA: Prepared the first draft of the manuscript. MSMR, RR, and MFN: The first draft was read, criticized, and corrected. MSMR and MFN: Proofread the second draft and finalized the manuscript. All authors have read and approved the final manuscript.
